# Health status transitions in community-living elderly with complex care needs: a latent class approach

**DOI:** 10.1186/1471-2318-9-6

**Published:** 2009-02-03

**Authors:** Louise Lafortune, François Béland, Howard Bergman, Joël Ankri

**Affiliations:** 1 Department of Health Administration, Université de Montréal, Québec, Canada; 2 Laboratoire Santé et Vieillissement, Université de Versailles-Saint-Quentin, INSERM U687, Paris, France; 3 Solidage Research Group, Montréal, Québec, Canada; 4 Division of Geriatric Medicine, McGill University, Jewish General Hospital, Montréal, Québec, Canada

## Abstract

**Background:**

For older persons with complex care needs, accounting for the variability and interdependency in how health dimensions manifest themselves is necessary to understand the dynamic of health status. Our objective is to test the hypothesis that a latent classification can capture this heterogeneity in a population of frail elderly persons living in the community. Based on a person-centered approach, the classification corresponds to substantively meaningful groups of individuals who present with a comparable constellation of health problems.

**Methods:**

Using data collected for the SIPA project, a system of integrated care for frail older people (n = 1164), we performed latent class analyses to identify homogenous categories of health status (i.e. health profiles) based on 17 indicators of prevalent health problems (chronic conditions; depression; cognition; functional and sensory limitations; instrumental, mobility and personal care disability) Then, we conducted latent transition analyses to study change in profile membership over 2 consecutive periods of 12 and 10 months, respectively. We modeled competing risks for mortality and lost to follow-up as absorbing states to avoid attrition biases.

**Results:**

We identified four health profiles that distinguish the physical and cognitive dimensions of health and capture severity along the disability dimension. The profiles are stable over time and robust to mortality and lost to follow-up attrition. The differentiated and gender-specific patterns of transition probabilities demonstrate the profiles' sensitivity to change in health status and unmasked the differential relationship of physical and cognitive domains with progression in disability.

**Conclusion:**

Our approach may prove useful at organization and policy levels where many issues call for classification of individuals into pragmatically meaningful groups. In dealing with attrition biases, our analytical strategy could provide critical information for the planning of longitudinal studies of aging. Combined, these findings address a central challenge in geriatrics by making the multidimensional and dynamic nature of health computationally tractable.

## Background

The general approach to studying older people's health has been to look at relationships among measures of chronic conditions, cognition, frailty and steps along the disablement pathway [1-4]; the goals have been to identify determinants and rates of decline and recovery [5-14], and predict mortality [15], institutionalization [16,13], and service utilization [17-21]. Although these measures serve as valid outcomes predictors, they relate differently to various dimensions of health. Trends in any one of them are not evidence of health trends overall [22,23]. Evidence suggests that population subgroups have qualitatively and quantitatively different patterns of change and probabilities of adverse outcomes [24,9,6,26,27,14,25,8]. In fact, elderly populations are highly heterogeneous in their health status owing to the variability and interdependency in how health dimensions manifest themselves overtime. Thus, it becomes increasingly clear that health changes cannot be fully described by any one dimension. Instead, it takes an approach that allows for multiple measures of health to embrace this complexity.

Many valid approaches exist to study the relationship between multiple health indicators and the unobservable (or latent) construct of health [28]. The choice depends, among other considerations, on the distribution of health indicators. Because health indicators commonly used in geriatrics are rarely continuous or normally distributed, factor analysis is unwieldy. Instead, we chose a latent class analytical framework [29], also called finite mixture models [30]. Latent class analysis (LCA) is a "person-centered" approach designed to divide the population under study into latent subpopulations (i.e. classes) that share a distinct interpretation pattern of relationships among indicators [31]. In populations of older people with complex health needs, observed health indicators can serve to identify groups of individuals – unobserved *a priori *– who present with similar constellations of health problems. The goal is to find the smallest number of health profiles that can describe the association among the set of observed health indicators.

Latent class models present several advantages over classical statistical models (e.g. cluster analysis) [32,33]: the classification is model-based and statistical diagnostic tools exist to assess the quality of the classification, variables may take several forms, there is no need to make parametric assumptions about the relationship between observations, and covariates can be included during model estimation to describe the groups. For the prediction of a dependant variable, LCA offer a parsimonious alternative to models with an unmanageably high number of variables and interaction terms.

Whereas LCA studies class membership using cross-sectional data, latent transition analysis (LTA) studies change in class membership using longitudinal data [31]. LTA combines the cross-sectional measurement of health state profiles and the description of transition over time from one health profile to another [34]. It thus supports the analysis of qualitative change in health status in contrast to continuous models were quantitative change is observed.

Several applications of LCA exist in the social, medical and economic literature, where heterogeneous populations constitute the typical object of analysis. In geriatrics, LCA was used to test criterion validity of physical frailty [35], measure mobility disability [36] and study behavioral syndromes in Alzheimer' patients [37]. We found no example of its use to model heterogeneity in older individuals' health status. Grade of membership (GoM), another latent classification technique [38], was successful in identifying clinically meaningful health profiles in community-living elderly [39-42]. GoM assumes that individuals can be partial members of more than one class of a continuous distribution of latent variables [43]. In contrast, LCA assumes that individuals are full members of one of the class for a discrete latent variable. Practically, LCA classifies individuals in health profiles whereas GoM estimates their degree of proximity to each profile. To our knowledge, these studies did not study transitions in profile membership; though Portrait et al. [41] studied mortality. Conversely, Markov models were used to describe transitions along the disablement pathway [14,9,8,44,45]. Although similar in spirit to LTA [34], these models focused on observable disability indicators as opposed to directly unobservable health status profiles.

The objective of this paper is to identify profiles of health status and study their evolution in a population of frail elderly individuals living in the community. We used LCA to group individuals into homogenous categories of health status based on observed indicators of prevalent health problems. We then applied LTA to study transitions in profile membership over 2 consecutive periods of 12- and 10-months, respectively. The question we address is whether there exists a latent classification that makes substantive sense to study the dynamic of health status in older populations presenting with complex care needs. Many research and policy questions call for a person-centered as opposed to a variable-centered approach [31]. Practical examples include planning of long-term care resources and identification of appropriate sub-groups for prevention studies. We identified and validated a classification that captures the multidimensional and dynamic nature of health, and show how a person-centered approach could prove useful to address these important questions.

## Methods

### Data sources

This research uses data collected for the randomized trial of the SIPA program (French acronym for System of Integrated Care for Older Persons), carried out in Montreal, Canada (1999–2001). SIPA aimed at improving continuity of community- and institution-based care for older people with complex health and social needs; its distinguishing features and the results of the experimentation are described elsewhere [46,47]. Eligible people were older than 64 years, competent in French or English (either the individual or the caregiver), had a score of -10 or less on the Functional Autonomy Measurement System (SMAF; -87 represents the worst health state) [48], and no plans for institutionalization within 3 months. Participants were recruited from two public community organizations responsible for home care (with a small proportion from other sources) and randomly assigned to the SIPA program or usual care. Health and sociodemographic data were collected via structured home interviews at baseline (T0), 12-months (T1), and 22-months (T2). Living status (deceased or not) and information on nursing home use come from administrative databases. Given the short intervention period, SIPA had no effect on change in health status or mortality [46]. For our purpose, we thus combined the two groups and included those who had a baseline questionnaire (n = 1164). The research ethics committee of the Montreal Jewish General Hospital granted approval for these secondary analyses.

#### Health indicators

LCA uses observed response patterns on a set of health indicators to model heterogeneity and reveal health profiles. We selected 17 indicators based on their high prevalence in older populations. Chronic conditions (no/yes) include self-reported hypertension, stroke, diabetes, cancer as well as circulatory, respiratory, joint and arthritis, stomach and bladder problems. To increase validity, individuals were asked whether a physician had confirmed the diagnosis. Sensory limitations (no/yes) refer to self-declared problems with speech, audition and/or vision. Cognition is measured with the Short Portable Mental Health Questionnaire (scores < 4 indicate no cognitive impairment; scores ≥4 indicate cognitive impairment) [49], and depression with the Geriatric Depression Scale (GDS scores < 3.5 indicate no depression; scores between 3.5–8.5 indicate moderate depression; scores ≥8.5 indicate severe depression) [50]. Functional limitations (no/yes) are defined as difficulty performing upper limbs (≥ 1; raising arms, picking/handling small objects, lifting 5 kg) and lower limbs (≥ 1; pulling/pushing large objects, bending/kneeling; using stairs) movements [51]. Disability is defined as requiring help with mobility activities of daily living (ADL) (getting up from bed/chair; using the toilet; taking a bath/shower; moving around the home, going up/down stairs, walking one block); personal care ADL (eating, drinking, dressing upper and lower body, personal grooming, washing) [52]; and instrumental ADL (IADL; using the phone, transportation, shopping, meal preparation, light housework, taking medication, managing money [53]. Disability measures are coded as three-levels categorical variables. The sociodemographic covariates used in our analyses are gender, age (64–75; 75–84; 85+ years) and living arrangements (whether people live alone or not).

### Analysis Strategy

LTA requires a step-wise approach to be accurate in its account of the change process. Using Mplus [54], we first explored alternative LCA models at (T0) to reveal the latent class variable that best captures the health status of our sample (i.e. number and characteristics of health profiles). Then, we fitted LCA models at (T1) and (T2) to examine the profiles' stability over time. Finally, we fitted LTA models to study transitions in health status.

#### Baseline health state profiles

Under latent class theory [55], individuals are assumed to belong to one of a number of unobserved categories (i.e. latent classes). Here, these categories represent health status profiles. It is assumed that a sufficient number of profiles result in conditional independence among observed health indicators [29]. In Mplus [54], LCA relies on maximum-likelihood methods to estimate the posterior probability p [c/Y] of membership in health state profile *c *for an individual with an observed health indicator response pattern *Y*. These posterior probabilities determine the relative prevalence and serve to characterize the profiles [28].

Two types of parameters serve to estimate class membership: health indicator and health profile probabilities. Health indicator probabilities are profile-specific and consist of the probability that a response is associated with the profile (e.g. probability of cognitive problems given membership in health profile 1). Within classes, individuals have comparable health indicator probabilities. Health profile probabilities represent individuals' probability of belonging to each profile; assignment to one profile proceeds on the basis of their highest health profile probability.

Using data for our 17 health indicators, we fitted LCA models successively for 1 through 5 classes. We used multiple start values to avoid convergence on local maxima [34] and assumed a missing at random mechanism (MAR), i.e. missingness depends on the observed components of the complete data and not on missing data [56]. For most health indicators, MAR is justified given the low proportion of missing data. For depression, however, the depression score is missing for all individuals with cognitive impairment (n = 174) and for those who are missing on the cognitive test (n = 101). To test the effect of the MAR hypothesis on the classification, LCA models were fitted with and without the depression indicator for 1) the complete sample (n = 1164), 2) a sub-sample with complete data for depression (n = 890) and 3) a sub-sample that excludes individuals with missing data for the cognition and depression scores (n = 1063). These sensitivity analyses showed that although depression significantly contributes to the classification, assuming a MAR mechanism for missing depression scores did not significantly change how individuals are grouped.

We decided on the best model based on the lowest values observed for the Akaike Information Criteria (AIC), Bayesian Information Criteria (BIC) and adjusted BIC (aBIC), which combine goodness of fit and parsimony [34]. The adjusted Lo-Mendell-Rubin likelihood ratio test (aLMR-LRT) served to decide on the number of classes [57]; it compares improvement in fit (p < 0.001) between sequential class models through an approximation of the LRT distribution. We used an entropy measure to assess how well the model predicts class membership; values range from 0 to 1 and high values are preferred [54]. Bivariate residual statistics served to confirm local independence [34].

Including covariates in LCA models can serve three purposes: describe the formation of health profiles, characterize and validate them. This is accomplished by the concurrent identification of the latent health profiles variable and its multinomial regression on covariates of interest [29,36]. This approach avoids the limits of post-hoc regressions (i.e. performed on *a priori *classified individuals), which assume that the latent classification is an observed variable measured without error [29]. We found that including gender, age and/or living arrangements as covariates did not significantly influence the formation of the profiles. Therefore, the LCA model without covariates is our basecase. LCA regression models are used for characterization and validation.

To further validate the profiles, we fitted 2 LCA models to estimate the association between profile membership and distal outcomes: mortality and use of nursing home services at 22-months. These associations were estimated by allowing the proportion for each outcome to vary across profiles [54]. Age and gender are included to control for confounding. Differences between classes are reported as odd ratios. Finally, to evaluate construct validity, we measured the relationship between class membership and disability measures (ADL-Personal care, ADL-Mobility, IADL), cognitive status and comorbidity (# chronic conditions) with chi-square and contingency coefficient statistics.

#### T1 and T2 health state profiles

Using available data, we proceeded the same way to identify health status profiles at subsequent time points. To assess whether the health status profiles identified at T1 and T2 have the same substantive meaning as those identified at baseline, we first compared patterns of health indicators probabilities over time. Then, we compared the classifications to those obtained by LCA models constrained to have baseline class-specific health indicator probabilities. The concordance (%) in how individuals are grouped is used to confirm the classification' stability over time. This also allows testing of the classification' robustness to death and lost to follow-up (LTF) attrition.

#### Latent transition analysis

Building from LCA, LTA studies change in class membership using longitudinal data [31,58]. Health indicators are measured repeatedly over time to identify profile membership at each occasion. The transition probability matrices are estimated by a logistic regression for nominal response (e.g. probability of health profile membership at T1, conditional on baseline health profile membership) [33,54]. When covariates are included in LTA, transition probabilities are no longer conditioned only on the previous time(s) membership but also on covariate values [59].

We ran two separate LTA: one to assess transitions in health status between T0 and T1 (LTA-T0T1); one to assess transitions between T1 and T2 (LTA-T1T2). Age and gender are included in our models – a provision that allows us to compare transition probabilities across these covariates. Death and LTF are modeled as absorbing states. Thus, each model adjusts the probability of health status transition for the competing risks of death and being LTF. Finally, we imposed measurement invariance (MI; constraining conditional health indicator probabilities to be the same across time points) to ensure health profiles have the same meaning at each occasion. The plausibility of MI was confirmed by comparing the classifications with and without MI constraints: the differences in classification were not significant (χ^2^; p < 0.001) and information criterion favored the constrained model.

## Results

### Sample description

The baseline sample consists of 1164 people between 64 and 104 years old, with 70.9% female and considerable socioeconomic variability (Table 1). Comorbidity ranges from none to seven chronic conditions. Comparisons of health indicators proportions reveal great heterogeneity in health status (Table 2). Given the low proportions of missing values, attribution under the MAR hypothesis had no effect on health indicator proportions, except for depression, cognition and bladder problems. For these indicators, Mplus modeled proportions appear in parentheses.

**Table 1:** Sample characteristics at T0; % of deceased and LTF individuals at T1 and T2 *

		**Baseline**	**T1**		**T2**	
		% (n = 1164)	**Deceased** % (n = 134)	**LTF** % (n = 232)^§^	**Deceased** % (n = 242)	**LTF** % (n = 446)

**Age**	Average (± SD)	82.2 (7.2)	**83.7 (7.6)**	82.2 (7.3)	**84.3 (7.2)**	82.1 (7.4)
	64–74 yrs	16.1	**12.7**	15.9	**9.5**	16.1
	75–84 yrs	43.8	**35.8**	41.4	**38.8**	42.0
	>84 yrs	40.2	**51.5**	42.7	**51.7**	41.9

**Gender**	Female	70.9	**56.0**	73.3	**57.4**	74.0
	Male	29.1	**44.0**	26.7	**42.6**	26.0
**Marital status**^**†**^	Married	33.2	**40.6**	34.3	**40.8**	30.4
	Not married	66.8	**59.4**	65.7	**59.2**	69.6
**Living arrangements**	Lives alone	43.7	**26.1**	46.8	**30.7**	46.7
	Not alone	56.6	**73.9**	53.2	**69.3**	53.3
**Education**	Primary	32.3	33.8	32.6	35.2	32.7
	Secondary	48.6	44.9	47.3	47.0	48.8
	Higher	19.1	21.3	20.1	17.8	18.5
**Income sufficiency**^**†**^	Sufficient	62.7	64.9	59.5	62.7	61.4
	Not sufficient	37.3	35.1	40.5	37.3	38.6
**Comorbidity**	0	29.1	31.6	33.8	28.8	31.2
	1–2	42.8	37.6	41.1	37.9	43.6
	3+	28.1	12.6	25.1	33.3	25.2
**SMAF **^**c**^	Average (± SD)	-23.5 (12.0)	**-29.0 (13.8)**	-23.3 (11.7)	**-27.9 (12.7)**	-23.5 (12.5)

* Statistical difference calculated using χ^2 ^statistics for categorical variables and logistic regression for age and SMAF; values in bold are significantly different at p < 0.001.

^§ ^Includes individuals who did not complete the interview (n = 88) at T1 but who did at T2.

^† ^Married includes having a common law spouse. Income sufficiency: Does your income currently satisfy your needs? Sufficient = very well or adequately; Not sufficient = with some difficulty, not very well or totally inadequate. SMAF: French acronym for Functional Autonomy Measurement System; a minimum score of -87 indicates the worst health state [48].

**Table 2:** Health indicator proportions (%) at baseline, 12 months and 22 months*

		**T0** (n = 1164)	**T1** (n = 797)	**T2** (n = 475)
**Cognitive problems**	Yes	28.6 (31.4)	24.1 (25.8)	33.3 (35.9)
	Missing	8.9 (-)	6.6 (-)	7.4 (-)
**Depression**	Moderate	29.1 (38.1)	21.0 (28.5)	17.7 (25.8)
	Severe	15.0 (19.7)	20.2 (27.5)	20.4 (29.8)
	Missing	23.6 (-) ^§^	26.6 (-)	31.6 (-)
**High blood pressure**	Yes	27.0	27.6	37.9
	Missing	0.9	0.5	0.8
**Circulatory problems**	Yes	39.3	40.5	44.0
	Missing	0.9	0.9	1.1
**Stroke**	Yes	20.6	23.2	25.5
	Missing	0.7	0.8	0.4
**Diabetes**	Yes	19.0	18.7	20.0
	Missing	0.9	0.9	-
**Respiratory problems**	Yes	25.3	25.5	26.1
	Missing	0.6	0.1	0.6
**Joint & Arthritis**	Yes	50.0	51.6	56.8
	Missing	0.9	0.5	0.6
**Tumor or Cancer**	Yes	17.4	17.8	18.1
	Missing	0.8	0.5	0.2
**Bladder problems**	Yes	31.1 (34.1)	29.1 (32.1)	25.7 (28.7)
	Missing	8.8 (-)	9.4 (-)	10.5 (-)
**Stomach problems**	Yes	26.1	24.8	27.6
	Missing	0.5	0.6	0.2
**Sensory problems**	Yes	24.4	23.5	28.4
	Missing	0.4	0.4	1.1
**Functional limits (U)**	Yes	56.7	75.3	78.9
	Missing	0.6	0.1	1.3
**Functional limits (L)**	Yes	75.0	62.1	67.6
	Missing	0.9	0.1	1.3
**ADL-Mobility Disability**	None	31.0	29.7	22.5
	1–2 activities	38.4	32.7	35.6
	+ 2 activities	30.6	37.5	41.5
	Missing	-	-	0.4
**ADL-Personal Care Disability**	None	58.4	63.2	57.7
	1–2 activities	22.2	16.1	16.0
	+ 2 activities	19.4	20.7	25.9
	Missing	-	-	0.4
**IADL Disability**	0–2 activities	35.6	32.7	31.2
	3–4 activities	26.6	29.0	21.5
	+4 activities	37.8	38.0	46.7
	Missing	-	-	0.6

* Entries in parentheses are the proportions generated by Mplus under the MAR hypothesis; proportions for other health indicators are not affected by missing values.

^§ ^Of these 23.6% (n = 274) missing GDS scores, 14% (n = 163) are missing by design for severely cognitively impaired individuals. Real missing scores represent 8.7% only. At T1 and T2, 18.4% (n = 147) and 13.1% (n = 62) respectively are missing by design.

ADL = Activity of daily living; IADL = Instrumental activity of daily living; U = upper limbs; L = lower limbs The upper and lower limbs distinction was made to capture variability in functional ability.

At 12-months (T1), 11.5% (n = 134) of the sample had died, 7.6% (n = 88) did not complete the interview and 12.4% (n = 144) were lost-to-follow-up (LTF). At 22-months (T2), 20.8% (n = 242) of the sample had died, 38.2% (n = 446) were LTF. Individuals who died were more likely to be male, older; they had worst functional scores and were less likely to live alone. In bivariate analyses, LTF individuals, either at T1 or T2, did not significantly differ from the rest of the sample on sociodemographic characteristics or baseline health status (i.e. IADL, ADL-personal care, cognition, depression, sensory deficits, functional limitations) except for ADL-mobility at T1 (χ^2^, p < 0.05) and sensory deficits at T2 (χ^2^, p < 0.05). In multivariable models, only ADL-mobility remained significantly associated with LTF status at T1.

### Health state profiles

LCA models estimated for each time point suggest that a 4-class solution provides the best overall fit and explanation of the observed health indicators frequencies. The classification identified at baseline was reproduced in the T1 and T2 LCA as well as in LTA. Accordingly, we describe the four health state profiles identified at baseline. We then present results for subsequent steps with reference to that basecase.

Table 3 presents model fit statistics for LCA models fitted at baseline, T1 and T2. At baseline, increasing the number of classes improved the classification up to the fifth class (i.e. the LMR-LRT are no longer significant). Information criterion statistics suggest the four-class model best fits the data. The quality of the classification for that model is high (entropy: 0.805), with no identification problem (condition number: 0.233) and no major violation of conditional independence. LCA performed at T1 and T2 also point to 4 health status profiles.

**Table 3:** Model fit statistics for latent class analysis at baseline, T1, T2

	**T0**				**T1**		**T2**	
	**2 classes**	**3 classes**	**4 classes**	**5 classes**	**4 classes**	**5 classes**	**4 classes**	**5 classes**

**Sequential model comparisons**	2 *vs*. 1 classes	3 *vs*. 2 classes	4 *vs*. 3 classes	5 *vs*. 4 classes	4 *vs*. 3 classes	5 *vs*. 4 classes	4 *vs*. 3 classes	5 *vs*. 4 classes
								
**LMR LRT**								
Log-likelihood value (c+1 classes)	13265.31	13595.67	12239.68	12093.26	8366.61	8119.41	4603.94	4449.05
								
-2 difference in log likelihood	1468.19	2711.98	292.85	107.55	494.40	108.374	309.774	77.03
p value	0.000	0.000	0.0006	0.307	0.000	0.286	0.000	0.198

**Adjusted LMR LRT**	1458.80	2375.46	290.97	106.86	490.03	107.64	265.99	76.43
p value	0.000	0.000	0.0006	0.309	0.000	0.289	0.000	0.198

**Information criterion**								
AIC	25148.43	24483.26	24192.52	24296.97	16244.82	16348.45	9072.11	9039.11
BIC	25365.96	24493.48	24207.69	24848.37	16258.87	16858.66	9426.66	9483.32
Adjusted BIC	25229.38	24487.13	24198.16	24502.15	16249.34	16512.53	9150.57	9137.40

**Entropy**	0.814	0.791	0.805	0.765	0.815	0.804	0.830	0.849
**Condition number**	0.0029	0.309	0.233	0.0012	0.0524	0.0014	0.0301	0.022

LMR-LRT = Lo-Mendell-Rubin Likelihood Ratio Test; AIC: Akaike Information Criterion; BIC: Bayesian Information Criterion.

Condition number = ratio of largest Eigen value to the smallest Eigen value for the Fisher information matrix. Values less than 10E^-09 ^indicate problem with model identification.

Table 4 presents profile-specific health indicators probabilities for the four latent classes (λ; presented as %). These probabilities express how individuals within a profile differ from those in other profiles at each time point. For example, at T0, the first two profiles are characterized by high probabilities of cognitive problems (λ = 0.687 and λ = 0.858, respectively), whereas the other two are not (λ = 0.078 and λ = 0.139). Profiles are assigned a label to substantiate these differences.

**Table 4:** Health indicators distribution and conditional probabilities per health profile *

	**Cog&Physic Impaired**			**Cognitively Impaired**			**Physically Impaired**			**Relatively Healthy**		
	**T0**	**T1**	**T2**	**T0**	**T1**	**T2**	**T0**	**T1**	**T2**	**T0**	**T1**	**T2**

**Cognition**	68.7	58.8	74.2	85.8	71.1	89.2	7.8	2.5	12.1	13.9	6.6	2.4
**Depression**												
Moderate	43.5	25.9	28.1	24.7	43.8	18.3	45.3	30.3	26.0	32.2	24.1	26.4
Severe	35.7	45.6	46.8	8.3	12.8	12.9	25.1	38.9	38.2	9.9	9.8	19.3
**Hypertension**	24.7	25.7	33.0	13.8	17.3	17.5	32.9	34.9	45.6	27.8	26.1	41.1
**Circulation**	45.9	46.5	48.7	12.7	22.5	25.8	50.8	55.4	60.5	31.4	27.1	38.0
**Stroke**	37.1	36.6	39.4	11.5	23.4	23.1	21.1	20.4	17.4	11.1	14.4	21.8
**Respiratory**	26.7	33.2	27.9	9.0	10.9	5.5	35.7	30.4	38.9	19.0	19.1	21.3
**Diabetes**	20.9	21.4	19.1	15.7	9.3	6.9	20.2	18.0	19.3	18.2	22.1	25.6
**Joint & Arthritis**	46.4	52.6	47.9	19.2	24.3	33.6	71.9	72.7	79.5	40.1	38.8	58.4
**Cancer**	14.0	17.0	19.4	11.9	10.2	11.5	20.0	21.5	17.1	20.3	17.7	20.6
**Bladder**	24.3	24.9	19.3	11.8	1.5	6.4	43.6	50.3	42.6	36.8	28.8	33.2
**Gastrointestinal**	23.2	23.7	27.1	16.4	7.4	12.6	32.6	32.0	32.9	24.7	25.9	30.2
**Sensory**	48.4	44.7	56.4	23.2	24.7	21.7	18.7	16.1	15.1	14.0	11.9	15.6
**Fx Limits (U)**	84.4	95.3	92.9	12.8	22.9	8.0	78.6	86.9	94.2	28.6	20.1	50.3
**Fx Limits (L)**	98.4	99.1	100	47.3	62.8	58.7	94.3	96.6	92.6	47.4	33.4	59.1
**ADL-Mobility**												
None	0.0	0.0	1.6	32.3	17.8	25	11.4	20.5	0.0	77.6	74.3	56.7
1–2 activities	14.7	7.5	8.3	57.2	54.8	59.9	60.5	49.9	49.3	22.4	23.5	41.6
+2 activities	85.3	92.5	90.0	10.4	27.5	15.1	28.1	29.6	50.7	0.0	0.0	1.8
**ADL-Personal care**												
None	7.1	11.0	12.0	57.9	56.5	75.1	60.8	79.5	52.2	95.1	96.1	96.2
1–2 activities	21.7	20.4	10.8	31.4	28.4	18.9	33.7	17.7	38.0	4.9	3.9	3.8
+ 2 activities	71.2	68.5	77.2	10.7	15.1	6.0	5.5	2.8	9.8	0.0	0.0	0.0
**IADL**	0.0	0.0	0.0	4.7	0.0	18.3	27.8	36.3	11.7	84.8	74.7	79.9
0–2 activities												
3–4 activities	2.6	10.4	1.0	20.0	25.0	9.8	55.0	49.8	53.9	13.8	23.5	18.3
+4 activities	97.4	89.6	99.0	75.4	75.0	72.0	17.2	14.0	34.3	1.4	1.8	1.8

* Entries represent profile-specific probabilities (λ) × 100 of reporting problems for the index health indicator.

ADL refers to difficulty with activities of daily living; IADL refers to difficulty with instrumental activities of daily living; Fx limits U = functional limitations with upper limbs; L = lower limbs The upper and lower limbs distinction was made to capture variability in functional ability.

All selected health indicators significantly contribute to the classification (p < 0.001). However, the pattern of relationships along the cognitive and physical dimensions best describes the profiles' distinguishing features. Severe cognitive and physical impairments characterize the first health profile. Individuals have high probabilities of cognitive disorders, chronic conditions, stroke, sensory problems, and functional limitations. Their high probabilities of disability in IADL, personal care and mobility ADL capture the severity and combined consequences of these problems. This group is labeled "Cognitively & physically impaired" (Cog&Physic-Imp) and represents 23% of the sample at baseline. The second health profile is predominantly "COGNITIVELY IMPAIRED" (Cog-Imp), with minimal physical impairments. These individuals (11.4%) report relatively low probabilities for chronic conditions and functional limitations. The likelihood for them to present with ADL disability is comparatively low but high for severe IADL disability.

Individuals in the third health status profile have the highest probabilities for chronic conditions, but no cognitive problem. They are very likely to report depression, functional limitations and mobility disability, but unlikely to require help for personal care. We labeled this group "PHYSICALLY IMPAIRED" (Physic-Imp). Finally, we found a "RELATIVELY HEALTHY" (R-Healthy) profile. It comprises older people who report comparatively less chronic conditions (circulatory problems, respiratory diseases, arthritis, depression; p < 0.01) and who manifest low probabilities of disability, functional limitations and cognitive disorders. The later two profiles represent, respectively, 35.6% and 29.9% of the sample at baseline.

Comparison across time points of profile-specific health indicator probabilities (Table 4) shows that the profiles revealed at T1 and T2 correspond to constellations of health problems equivalent to those observed at baseline. When we compared T1 and T2 profiles with those obtained with T1 and T2 models constrained to have baseline conditional health indicator probabilities, the concordance in how individuals are grouped reaches 90% at T1, and 88% at T2. At T1, the concordance is 96% for the Cog&Physic-Imp, 92% for the Cog-Imp, 83% (p < 0.01) for the Physic-Imp and 86% (p < 0.05) for the R-Healthy. At T2, the concordance is 83% (p < 0.05) for the Cog&Physic-Imp (with 13% classified as Physic-Imp), 100% for the Cog-Imp, 69% (p < 0.001) for the Physic-Imp (with 27.4% classified as R-Healthy), and 95.6% for the R-Healthy. These differences in classification reflect the net progression of the sample as a whole towards a more compromised health state (as seen in tables 2 and 4). Although this results in an upward shift in the "severity" of health profiles at T1 and T2, each maintained its substantive meaning. Combined, these results confirm the stability of our classification, despite mortality and LTF.

The health indicator probabilities predicted by LCA are consistent with the proportion of people who have contributed each response patterns in the actual data. Furthermore, chi-square and contingency coefficients (Table 5) for health state profiles versus disability measures, comorbidity and cognitive problems confirm both the qualitative differences between health profiles and their coherence with key measures of health status. The correlation with the comorbidity measure is much lower.

**Table 5:** Health indicators' prevalence by health status profile at T0 (%)

		**Cog&Physic Imp**	**Cog-Imp**	**Physic-Imp**	**R-Healthy**
**ADL-Mobility** n = 1164	No	0	35.1	9.5	79.3
	1–2 activities	14.1	56.7	63.3	20.7
	+ 2 activities	85.9	8.2	27.2	0
	χ^2^ (p-value)		955.01 (p < 0.001)		
	Adjusted C*		0.822 (p < 0.001)		

**ADL-Personal care** n = 1164	No	6.3	59.0	60.9	95.7
	1–2 activities	23.0	31.3	33.7	4.2
	+ 2 activities	70.7	9.7	5.3	0
	χ^2^ (p-value)		770.27 (p < 0.001)		
	Adjusted C		0.773 (p < 0.001)		

**IADL** n = 1164	0–2 activities	0	2.2	26.2	87.3
	3–4 activities	1.1	19.4	57.5	12.4
	+4 activities	98.9	78.4	16.3	0.3
	χ^2 ^(p-value)		1156.42 (p < 0.001)		
	Adjusted C		0.865 (p < 0.001)		

**Cognitive problems** n = 1159	None	30.4	10.7	92.9	86.1
	Moderate	6.5	13.2	3.9	8.8
	Severe	63.1	76.0	3.1	5
	χ^2^ (p-value)		556.05 (p < 0.001)		
	Adjusted C		0.719 (p < 0.001)		

**Comorbidity** n = 1158	0	25.4	71.4	11.9	36.1
	1–2	48.9	24.1	45.3	42.5
	>3	25.7	4.5	42.8	21.4
	χ^2^ (p-value)		208.70 (p < 0.001)		
	Adjusted C		0.479 (p < 0.0001)		

* C = Contingency coefficient with adjustment so it reaches a maximum of 1 (i.e. C/Sqr-root of k1-/k, where k is the number of rows or column, whichever is less)

ADL refers to difficulty with activities of daily living; IADL refers to difficulty with instrumental activities of daily living

The relationships between profile membership, covariates, and distal outcomes also support the validity of the classes. Results of LCA regression models indicate that Cog&Physic-Imp individuals are significantly older compared to those classified in the Physic-Imp and R-Healthy profiles. Relative to their younger peers (64–74 years), individuals in the age groups 75–84 and 85+ are 1.6 and 2.5 times more likely to be highly disabled as opposed to being "only" physically impaired (p < 0.01). These odds increase to 2 and 4, respectively, when compared to being relatively healthy (p < 0.001). Whereas women tend to be classified in the profiles with disability or be relatively healthy, men are significantly more likely to be classified in the Physic-Imp profile (OR range: 2.18–3.2; p < 0.01), where the probability of any type of disability is comparatively low despite high probability of chronic conditions. In both profiles characterized by cognitive problems, individuals are more likely not to live alone (OR range: 3.84–9.28; p < 0.001).

Controlling for age and gender, we found that Cog&Physic-Imp individuals are significantly more likely to die within 22-months compared to Cog-Imp (OR 2.84; p < 0.002), Physic-Imp (OR 3.27; p < 0.001) and R-Healthy individuals (OR 4.75; p < 0.001). In turn, Cog-Imp individuals are more likely to die compared to the Physic-Imp (OR 1.15; p < 0.003) and the R-Healthy (OR 1.67; p < 0.003); and Physic-Imp individuals more likely to die than the R-Healthy (OR 1.45; p < 0.001). Finally, we find that Cog&Physic-Imp individuals are less likely to use nursing home services within 22-months compared to Cog-Imp individuals (OR 0.72; p < 0.001). In turn, individuals in the later two profiles, i.e. characterized by cognitive impairments, are more likely to use nursing home services compared to Physic-Imp individuals (OR 2.87 and 4.37, respectively; p < 0.001) and R-Healthy individuals (OR 3.18 and 4.38, respectively; p < 0.001). The likelihood of using nursing home services for Physic-Imp relative to the R-healthy is not as marked (OR 1.11; p < 0.004). These results all point in the expected direction.

### Latent transition analyses

Table 6 presents age-controlled latent transition probabilities from baseline to T1 and from T1 to T2. Among Cog&Physic-Imp individuals, 51.4% are predicted to remain in that state and 24.9% are predicted to die within a year. Around 5% are predicted to move towards less disabled health states: 3.0% towards a Cog-Imp state and 2.3% towards a Physic-Imp state. The main gender difference lies in the increased probability of death for men and LTF for women. Among Cog-Imp individuals, women are more likely to transition towards the more disabled state (24.1%) or improve (4%), whereas men are comparatively more likely to stay in their same state (47.8% *vs*. 24.3%) or die (11.2% *vs*. 9%). The competing risk for women to be LTF is again higher. Physic-Imp individuals are characterized by more stability (63.8%) with no striking gender difference. Overall, their probability of transitioning towards more disabled states is 4.3%, and somewhat higher for men; their transition probability towards the R-healthy state is 3.4%, and somewhat higher for women. Men are twice as likely to die. Although the R-Healthy also tend to be characterized by stability (66.2%), this applies most importantly to women. Over a year, men' transition probabilities towards more compromised health states is higher despite their higher competing risk for dying or being LTF.

**Table 6:** Transition probabilities *

	**12 months**					
	**Cog&Physic-Imp**	**Cog-Imp**	**Physic-Imp**	**R-Healthy**	**Deceased**	**LTF**
**Baseline**						
**Cog&Physic-Imp**	**0.514**	**0.030**	**0.023**	**0.000**	**0.249**	**0.184**
Female	0.550	0.013	0.033	0.000	0.184	0.220
Male	0.502	0.032	0.025	0.000	0.284	0.156
**Cog-Imp**	**0.181**	**0.438**	**0.001**	**0.023**	**0.101**	**0.256**
Female	0.241	0.243	0.001	0.040	0.090	0.385
Male	0.177	0.478	0.001	0.013	0.112	0.219
**Physic-Imp**	**0.043**	**0.000**	**0.638**	**0.034**	**0.064**	**0.221**
Female	0.036	0.000	0.692	0.033	0.040	0.199
Male	0.042	0.000	0.683	0.017	0.079	0.179
**R-Healthy**	**0.046**	**0.025**	**0.041**	**0.662**	**0.071**	**0.155**
Female	0.037	0.008	0.042	0.732	0.038	0.143
Male	0.061	0.036	0.059	0.551	0.107	0.185
						
	**22-months**					
	**Cog&Physic-Imp**	**Cog-Imp**	**Physic-Imp**	**R-Healthy**	**Deceased**	**LTF**

**12-months**						
**Cog&Physic-Imp**	**0.412**	**0.013**	**0.000**	**0.000**	**0.153**	**0.422**
Female	0.477	0.006	0.000	0.000	0.104	0.413
Male	0.402	0.019	0.000	0.000	0.196	0.384
**Cog-Imp**	**0.256**	**0.329**	**0.000**	**0.018**	**0.037**	**0.361**
Female	0.353	0.171	0.000	0.028	0.029	0.420
Male	0.224	0.422	0.000	0.020	0.040	0.294
**Physic-Imp**	**0.062**	**0.003**	**0.479**	**0.022**	**0.076**	**0.358**
Female	0.065	0.001	0.521	0.025	0.055	0.333
Male	0.070	0.005	0.363	0.030	0.132	0.399
**R-Healthy**	**0.062**	**0.049**	**0.101**	**0.406**	**0.013**	**0.369**
Female	0.064	0.019	0.111	0.467	0.009	0.330
Male	0.057	0.065	0.064	0.471	0.017	0.326

*The overall transitions control for age and gender. Female and male transition probabilities represent marginal probabilities, also controlling for age. LTA ran under the assumption of MI.

The patterns of transitions between T1 to T2 are similar, with quantitative differences. Overall, transition probabilities towards more disabled states are higher, and improvements less likely. A noteworthy gender difference is the increased probability of R-Healthy women to transition to the Physic-Imp or Cog-Physic-Imp states, as opposed to stability. Also, Cog-Imp women have a 35% probability of becoming highly disabled whereas men in that state appear more stable. The probability of dying across health profiles is lower, except for Physic-Imp men. These differences must be considered in light of the increased competing risk of being LTF and the 10-month interval.

## Discussion

Our aim was to identify a meaningful latent classification that encompasses multiple dimensions of health and captures their synergistic effect on older people's health status. We identified four homogeneous health state profiles that are stable over time and sensitive to change.

The uncovered classification has face validity. It clearly distinguishes the physical and cognitive dimensions of health. And within each of these dimensions, a qualitative distinction along the disability dimension captures the consequences of diseases and impairments [1]. These findings generally agree with classifications obtained by other methods [39,41,40,42]. In elderly populations comparable to ours [39,40], published classifications revealed more nuanced groups (i.e. 5–7 profiles) but with meanings anchored in the same dimensions as those characterizing our profiles. In samples representative of community-living older people [41,42], an additional "Healthy" profile typically emerges. Given our target population's compromised health, we did not find nor expected a healthy profile. In our sample, four latent classes were sufficient to capture health status heterogeneity while maintaining interpretability and stability over time. There is no longitudinal evidence to determine whether the additional profiles of previously published classifications possess the later qualities.

The profiles' stability over and above observed changes in the sample's overall health substantiates the validity of our classification. The profiles are robust not only in being comparable across time points but also in holding despite high mortality and LTF. The differentiated and gender-specific patterns of transition probabilities demonstrate the profiles' sensitivity to change in health states. Although most individuals tended to remain in their health state or died, we found higher probabilities of unfavorable transitions for individuals in the more compromised health states, and lower probabilities of improvements. These observations concord with studies of change in disability [7,26,25], functional limitations [6,11,10] and frailty [60]. The consistent finding, across outcome measures, is a decreased probability of recovery and an increased probability of decline or death when more deficits are reported at baseline. For the Cog-Imp profile, our findings appear consistent with the course of disability progression as cognitive problems worsen: IADL are affected first, followed by basic activities of personal care [4,6]. Yet, a shortcoming of traditional functional measures is their inadequate ability to detect cognitive impairments, particularly when scaled with items influenced by physical ability [61]. Our results show that classification into homogenous health categories unmasked the differential relationship of physical and cognitive domains with progression in disability; gender specific analyses provide further insights.

Overall, the classification has good construct validity. We observed higher mortality and older individuals in the more vulnerable groups; an increased likelihood of nursing home use for cognitively impaired profiles; gender differences in transition probabilities, as well as high coherence between class membership and individual health indicators except comorbidity. The latter is consistent with previous work showing that assessment of disease alone is a weak marker of health status in older individuals, even when indicators of disease severity are considered [62].

In considering the generalizability of our findings, it is important to keep in mind that our reference population was selected to demonstrate the value of integrated services for older people with complex care needs [46,47]. The characteristics of our sample thus closely match those of the sub-population of community-living elderly targeted by such programs [63], not those of the general population; our classification reflects their compromised health status. Evidence shows that particular groups, namely frail elderly, may be more likely than others to benefit from better integration of care [46] but identifying them remains a challenge. Application of LCA may prove useful for doing so.

Three other issues deserve discussion. The first relates to missing depression and cognitive scores. Firstly, depression scores are mainly missing by design for the cognitively impaired. Assuming a MAR mechanism for depression did not significantly change how individuals are grouped yet we cannot exclude misclassification for individuals who also have a missing cognition score. Secondly, despite the known association between depressive symptoms and cognitive impairments [64], we could not assess the effect of our MAR assumption on subsequent cognitive decline.

The second issue pertains to attrition. LTF individuals did not differ on sociodemographic characteristics and most health indicators at baseline but differed on mobility disability. Combined with our inability to control for unobserved individuals effects (e.g. lifestyle, social support), this means that we cannot exclude a selection bias. Yet, there is no significant difference between profiles in the proportions of LTF individuals at T1 (χ^2^:6.317; p = 0.097) or T2 (χ^2^:2.544; p = 0.467). Moreover, the high concordance in how individuals are grouped confirmed the classification's stability overtime, which also points to its robustness to the competing risk of being LTF. For the transition analyses, we captured LTF individuals through an absorbing state. This provision does not inform us on the effect of change in health status on attrition – or *vice versa*. Nevertheless, it deals with the potential attrition biases introduced in transition studies when these individuals are excluded.

Conversely, a mortality bias is unlikely: we recorded death using administrative databases and captured these transitions through an absorbing state. Excluding deceased individuals would have yielded a healthier sample, overestimated stability and recovery, and underestimated progression relative to a more representative sample. Our modeling approach avoids this bias without having to modify the indicators, run separate analyses or use imputation techniques – all common shortcomings in geriatric studies [65,66].

Thirdly, we performed the transition analyses in two steps to avoid convergence problems due to the large number of missing data patterns. This approach is not as powerful as performing one LTA on 3 time points but it allowed us to account for the competing risks of death and being LTF in the same analyses. Moreover, because we constrained health profiles to have the same meaning across time points, this two-step strategy should yield valid transition patterns. To be sure, concurrent information on transition, death and LTF probabilities by health status provides critical information for the planning of longitudinal studies of aging.

Despite those limits, our work tackles a core challenge of gerontology research by making the multidimensional and dynamic nature of older people's health status computationally tractable. LCA capture multiple dimensions of health; reveal the smallest number of health profiles that can explain away the associations among observed health dimensions; and makes no assumption about the distribution of health indicators or their relationships other than that of local independence [34]. On these methodological grounds, LCA supersedes classical statistical models by eliminating part of the endogeneity bias [67] introduced in multivariable modeling when indicators of diseases, cognition and disability enter in the model simultaneously. Dealing with this problem is even more pressing when measuring the dynamic of health status, which implies concomitant and interrelated changes in various factors over time [68]. LTA provides a useful empirical heuristic for studying this complex process because the measurement model is specifically developed for dynamic variables as an outgrowth of substantive theory [33,69].

## Conclusion

In his seminal paper on the compression of morbidity, Fries argues that the means for affecting positive change in an aging population are to be found in the variability of the population, as well as in the average values [70]. Our study presents some means to identify and quantify inter-individual variability in health status. Notably, the important weight of the cognitive dimension in explaining this variability and transitions along the disability dimension underscores the importance of moving beyond "simple" functional measures if we are to comprehend the dynamic of elderly people health and social needs. The combination of chronic conditions, cognition and disability items for our LCA finds a parallel in the approaches used to develop the Frailty Index [71] and Clinical Frailty Scale [72]. Compared to our profiles, the former continuous measures of health status provide finer gradations likely to be pertinent to clinical practice and aging research. Conversely, our approach may be unwieldy for clinical use but finds its application at organization and policy levels where many issues call for classification of individuals into pragmatically meaningful groups. Econometric modeling has already demonstrated the sensitivity of such classifications to differences, and changes, in available patterns of health and social services in specific milieu [40,73]. Applications of LCA and LTA to larger, more representative samples are needed to confirm our findings and expand the methodological underpinnings of these approaches to study health status in older populations.

## Abbreviations

**ADL**: Activity of Daily Living; **AIC**: Akaike Information Criteria; **BIC**: Bayesian Information Criteria; **Cog-Imp**: Cognitively Impaired; **Cog&Physic-Imp**: Cognitively and Physically Impaired; **GDS**: Geriatric Depression Scale; **GoM**: Grade of Membership; **IADL**: Instrumental Activity of Daily Living; **LCA**: Latent Class Analysis; **LMR-LRT**: Lo-Mendell-Rubin Likelihood Ratio Test; **LTF**: Lost to Follow-up; **LTA**: Latent Transition Analysis; **MAR**: Missing at Random; **Physic-Imp**: Physically Impaired; **R-Healthy**: Relatively Healthy; **SIPA**: System of Integrated Care for Older People.

## Competing interests

The authors declare that they have no competing interests.

## Authors' contributions

LL conceived and designed the study, performed the statistical analyses, drafted and revised the manuscript. FB made substantial contributions to the conception and design of the study, acquisition of the original data and interpretation of the data. HB made substantial contributions to the acquisition of the original data. FB, HB and JA all critically revised the manuscript. All authors read and approved the final manuscript.

## Pre-publication history

The pre-publication history for this paper can be accessed here:


